# Tris(1*H*-benzimidazol-3-ium-2-ylmeth­yl)amine tris­(2,4,6-trinitro­phenolate) acetonitrile disolvate

**DOI:** 10.1107/S160053681202329X

**Published:** 2012-05-31

**Authors:** Ying Bai, Jing-Kun Yuan, Hua Wang, Guo-Long Pan, Hui-Lu Wu

**Affiliations:** a School of Chemical and Biological Engineering, Lanzhou Jiaotong University, Lanzhou 730070, People’s Republic of China

## Abstract

In the cation of the title salt, C_24_H_24_N_7_^3+^·3C_6_H_2_N_3_O_7_^−^·2C_2_H_3_N, the three benzimidazolium ring systems are oriented to each other at dihedral angles of 10.42 (7), 23.98 (7) and 22.17 (7)°. In the crystal, the cation links to the adjacent picrate anions *via* N—H⋯O hydrogen bonds; one of independent acetonitrile solvent mol­ecules is also linked to the cation *via* an N—H⋯N hydrogen bond.

## Related literature

For background to benzimidazoles and their derivatives, see: Wilkinson (1987 ▶); Siya *et al.* (1992 ▶); Horton *et al.* (2003 ▶); Prados & Quesada (2008 ▶); Steed (2009 ▶); Aghabozorg *et al.* (2008 ▶). For inter­molecular inter­actions, see: Blake *et al.* (2000 ▶); Bourne *et al.* (2001 ▶); Desiraju (2000 ▶). For our previous model studies, see: Liu *et al.* (2011 ▶);
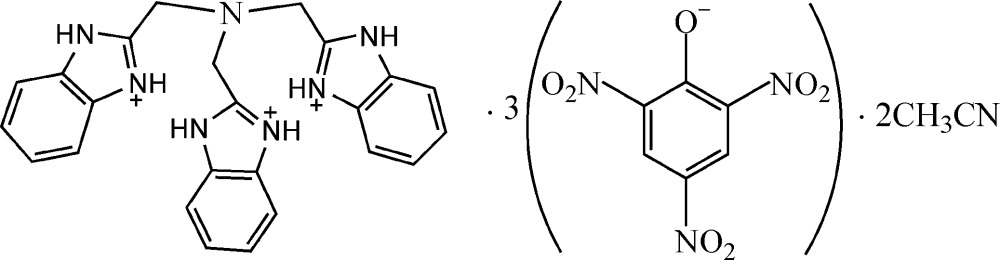


## Experimental

### 

#### Crystal data

C_24_H_24_N_7_^3+^·3C_6_H_2_N_3_O_7_^−^·2C_2_H_3_N
*M* *_r_* = 1176.93
Triclinic, 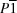

*a* = 10.9914 (3) Å
*b* = 15.4620 (5) Å
*c* = 16.1760 (6) Å
α = 74.826 (1)°
β = 74.337 (1)°
γ = 73.299 (1)°
*V* = 2484.29 (14) Å^3^
*Z* = 2
Mo *K*α radiation
μ = 0.13 mm^−1^
*T* = 153 K
0.38 × 0.36 × 0.30 mm

#### Data collection

Bruker APEXII CCD diffractometer
18903 measured reflections
8608 independent reflections
6896 reflections with *I* > 2σ(*I*)
*R*_int_ = 0.016

#### Refinement

*R*[*F* ^2^ > 2σ(*F* ^2^)] = 0.037
*wR*(*F* ^2^) = 0.127
*S* = 1.14
8608 reflections
769 parameters
H-atom parameters constrained
Δρ_max_ = 0.60 e Å^−3^
Δρ_min_ = −0.52 e Å^−3^

### 

Data collection: *APEX2* (Bruker, 2007 ▶); cell refinement: *SAINT* (Bruker, 2007 ▶); data reduction: *SAINT*; program(s) used to solve structure: *SHELXS97* (Sheldrick, 2008 ▶); program(s) used to refine structure: *SHELXL97* (Sheldrick, 2008 ▶); molecular graphics: *SHELXTL* (Sheldrick, 2008 ▶); software used to prepare material for publication: *SHELXTL*.

## Supplementary Material

Crystal structure: contains datablock(s) global, I. DOI: 10.1107/S160053681202329X/xu5538sup1.cif

Structure factors: contains datablock(s) I. DOI: 10.1107/S160053681202329X/xu5538Isup2.hkl

Supplementary material file. DOI: 10.1107/S160053681202329X/xu5538Isup3.cml

Additional supplementary materials:  crystallographic information; 3D view; checkCIF report

## Figures and Tables

**Table 1:** Hydrogen-bond geometry (Å, °)

*D*—H⋯*A*	*D*—H	H⋯*A*	*D*⋯*A*	*D*—H⋯*A*
N1—H1*A*⋯O1	0.88	1.96	2.834 (2)	173
N2—H2*A*⋯O15^i^	0.88	1.88	2.619 (2)	140
N2—H2*A*⋯O16^i^	0.88	2.27	2.952 (2)	134
N3—H3*A*⋯O1	0.88	2.01	2.847 (2)	158
N3—H3*A*⋯O7	0.88	2.33	2.905 (2)	123
N4—H4*A*⋯N17^ii^	0.88	2.18	2.965 (3)	148
N5—H5*A*⋯O1	0.88	2.28	2.836 (2)	121
N5—H5*A*⋯O2	0.88	2.09	2.853 (2)	144
N6—H6*A*⋯O8^iii^	0.88	1.91	2.693 (2)	147
N6—H6*A*⋯O14^iii^	0.88	2.30	2.945 (2)	130

Symmetry codes: (i) 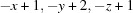; (ii) 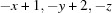; (iii) 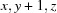.

**Fig. 1.**
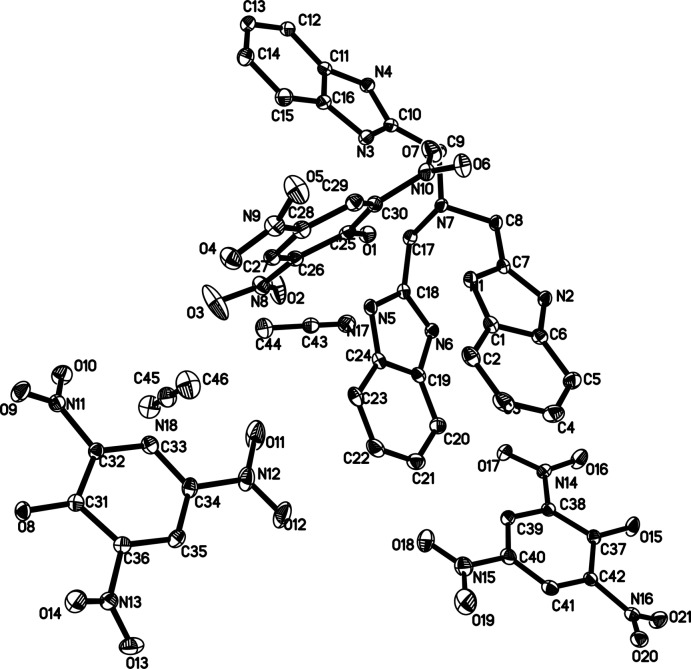
The title compound with displacement ellipsoides drawn at the 30% probability level. H atoms bonded to C atoms have been omitted for clarity.

## supplementary crystallographic information

### Comment

It is well known that benzimidazole is a typical heterocyclic ligand with nitrogen donor and a component of biologically important molecules (Wilkinson *et al.*, 1987). Those compounds are environmentally friendly compounds with two high active nitrogen atoms in 1, 3-sites (Siya *et al.* 1992). Benzimidazoles and their derivatives being ubiquitous, quite a few of them play important roles in biological, aquatic, environmental, and industrial processes, fungicide and many other fields (Horton *et al.*, 2003; Steed, 2009; Prados & Quesada, 2008). According to the previous report (Aghabozorg *et al.*, 2008), H. Aghabozorg *et al.* focused on the proton delivery from acids, which are considered as suitable proton donors, to amines as proton acceptors. The results were production of several proton transfer ion pairs possessing some remaining donor sites applied for coordination to metallic centers in preparation of metal-organic structures. Much of the investigations show that the proton compound exist various interactions including hydrogen bondings, ion pairing, van der Waals and so on (Bourne *et al.*, 2001; Desiraju *et al.*, 2000; Blake *et al.*, 2000).

In our previous model studies (Liu *et al.*, 2011) that the bis(*N*-methylbenzimidazol-2-ylmethyl) aniline (MEBBA) cation attacked by a picrate anion bridge with proton transfer and formation of a novel complex, now we used similar method to synthesize the title compound. The title compound is a proton transfer compound that consists of a tris (2-benzimidazolylmethyl) amine cation, three picrate anions and diacetonitrile solvents. Three protons from three picrate anion transfer to N (double bond) fromtris (2-benzimidazolylmethyl) amine cation. The proton transfer compound is formed by picrate anions and amines can enhance the intermolecular forces between the obtained cationic and anionic fragments, and interactions described above can provide a large part of the stabilization energy of resulting self-assembly systems (Aghabozorg *et al.*, 2008). The crystal structure is mainly stabilized by N—H—N intramolecular hydrogen bond. In this paper, the crystal unit of the title proton transfer compound be composed and the proton of the picric acid is transferred to the nitrogen atoms of the ntb (Fig. 1), and formed by tripod structrue. The angle of C9–N7–C17 is 108.15°, C8–N17–C7 is 112.55°, C8–N7–C9 is 110.46° respectively. From the crystal structure we can see that there is one ntb ligand containing three N—H bonds as hydrogen-bonding donors, each forming an N—H—O hydrogen bond with the surrounding picrate anions.

### Experimental

Reagents and solvents used were of commercially available quality. To a stirred solution of tris (2-benzimidazolylmethyl)amine (0.4070 g, 1 mmol) in hot acetonitrile (10 ml) was added picrate acid (0.2291 g, 1 mmol) solution dissolved in acetonitrile (5 ml) over 4-5 h at room temperature, then the clear filtrate was collected from the resulting solution. The crystallized corresponding products were obtained from the filtrates by allowing slow evaporation of the solvent at room temperaturethe. (Yield 0.401 g, 63%). Elemental analysis found: C, 47.02%; H, 3.02%; N, 21.37%; calcd. for C46H36N81O21: C, 46.95%; H, 3.08%; N,21.42%.

### Refinement

H atoms were found in difference electron maps and were subsequently refined in a riding-model approximation with C—H = 0.95 to 0.99 Å and N—H = 0.88 Å, *U*_iso_(H) = 1.2*U*_eq_(C,N).

### Crystal data

**Table:**

C_24_H_24_N_7_^3+^·3C_6_H_2_N_3_O_7_^−^·2C_2_H_3_N	*Z* = 2
*M**_r_* = 1176.93	*F*(000) = 1212
Triclinic, *P*1	*D*_x_ = 1.573 Mg m^−^^3^
Hall symbol: -P 1	Mo *K*α radiation, λ = 0.71073 Å
*a* = 10.9914 (3) Å	Cell parameters from 8608 reflections
*b* = 15.4620 (5) Å	θ = 3.0–25.0°
*c* = 16.1760 (6) Å	µ = 0.13 mm^−^^1^
α = 74.826 (1)°	*T* = 153 K
β = 74.337 (1)°	Block, yellow
γ = 73.299 (1)°	0.38 × 0.36 × 0.30 mm
*V* = 2484.29 (14) Å^3^	

### Data collection

**Table:**

Bruker APEXII CCD diffractometer	6896 reflections with *I* > 2σ(*I*)
Radiation source: fine-focus sealed tube	*R*_int_ = 0.016
Graphite monochromator	θ_max_ = 25.0°, θ_min_ = 3.0°
ω scans	*h* = −13→13
18903 measured reflections	*k* = −18→18
8608 independent reflections	*l* = −19→19

### Refinement

**Table:**

Refinement on *F*^2^	Secondary atom site location: difference Fourier map
Least-squares matrix: full	Hydrogen site location: inferred from neighbouring sites
*R*[*F*^2^ > 2σ(*F*^2^)] = 0.037	H-atom parameters constrained
*wR*(*F*^2^) = 0.127	*w* = 1/[σ^2^(*F*_o_^2^) + (0.070*P*)^2^ + 0.7354*P*] where *P* = (*F*_o_^2^ + 2*F*_c_^2^)/3
*S* = 1.14	(Δ/σ)_max_ = 0.003
8608 reflections	Δρ_max_ = 0.60 e Å^−^^3^
769 parameters	Δρ_min_ = −0.52 e Å^−^^3^
0 restraints	Extinction correction: *SHELXL97* (Sheldrick, 2008), Fc^*^=kFc[1+0.001xFc^2^λ^3^/sin(2θ)]^-1/4^
Primary atom site location: structure-invariant direct methods	Extinction coefficient: 0.0048 (7)

### Special details

**Table:**

Geometry. All e.s.d.'s (except the e.s.d. in the dihedral angle between two l.s. planes) are estimated using the full covariance matrix. The cell e.s.d.'s are taken into account individually in the estimation of e.s.d.'s in distances, angles and torsion angles; correlations between e.s.d.'s in cell parameters are only used when they are defined by crystal symmetry. An approximate (isotropic) treatment of cell e.s.d.'s is used for estimating e.s.d.'s involving l.s. planes.
Refinement. Refinement of *F*^2^ against ALL reflections. The weighted *R*-factor *wR* and goodness of fit *S* are based on *F*^2^, conventional *R*-factors *R* are based on *F*, with *F* set to zero for negative *F*^2^. The threshold expression of *F*^2^ > σ(*F*^2^) is used only for calculating *R*-factors(gt) *etc*. and is not relevant to the choice of reflections for refinement. *R*-factors based on *F*^2^ are statistically about twice as large as those based on *F*, and *R*- factors based on ALL data will be even larger.

### Fractional atomic coordinates and isotropic or equivalent isotropic displacement parameters (Å^2^)

**Table:**

	*x*	*y*	*z*	*U*_iso_*/*U*_eq_	
O1	0.19492 (13)	0.72945 (9)	0.17343 (9)	0.0277 (3)	
O2	0.39722 (16)	0.60052 (13)	0.11588 (14)	0.0557 (5)	
O3	0.36658 (17)	0.47442 (13)	0.10886 (18)	0.0768 (7)	
O4	−0.03082 (14)	0.38847 (10)	0.21627 (11)	0.0376 (4)	
O5	−0.20832 (15)	0.49423 (12)	0.23218 (13)	0.0488 (5)	
O6	−0.18403 (14)	0.78337 (11)	0.28475 (10)	0.0373 (4)	
O7	−0.04156 (14)	0.84451 (10)	0.18413 (11)	0.0387 (4)	
O8	0.55319 (13)	−0.01411 (9)	0.21443 (9)	0.0287 (3)	
O9	0.45012 (15)	0.11421 (11)	0.08342 (10)	0.0382 (4)	
O10	0.54969 (15)	0.22461 (11)	0.04584 (10)	0.0368 (4)	
O11	0.4936 (2)	0.37383 (13)	0.28626 (13)	0.0709 (6)	
O12	0.5011 (2)	0.29227 (13)	0.41583 (12)	0.0557 (5)	
O13	0.61627 (14)	−0.04480 (11)	0.44910 (10)	0.0412 (4)	
O14	0.49248 (17)	−0.08665 (11)	0.39075 (10)	0.0435 (4)	
O15	0.80096 (18)	0.92619 (10)	0.63659 (10)	0.0455 (4)	
O16	0.86187 (18)	0.99626 (11)	0.46725 (10)	0.0468 (4)	
O17	0.91497 (14)	0.90721 (11)	0.37525 (9)	0.0365 (4)	
O18	0.8705 (2)	0.59992 (13)	0.45699 (13)	0.0583 (5)	
O19	0.8206 (2)	0.53622 (13)	0.59356 (15)	0.0723 (6)	
O20	0.74359 (14)	0.70416 (10)	0.81857 (10)	0.0343 (4)	
O21	0.84323 (16)	0.81413 (11)	0.78892 (10)	0.0402 (4)	
N1	0.19365 (15)	0.80823 (11)	0.31345 (11)	0.0235 (4)	
H1A	0.1904	0.7887	0.2678	0.028*	
N2	0.20614 (15)	0.89781 (11)	0.39250 (10)	0.0238 (4)	
H2A	0.2129	0.9467	0.4075	0.029*	
N3	0.16194 (15)	0.88274 (11)	0.03191 (11)	0.0244 (4)	
H3A	0.1554	0.8451	0.0833	0.029*	
N4	0.19477 (15)	1.00153 (11)	−0.06998 (10)	0.0236 (4)	
H4A	0.2132	1.0551	−0.0966	0.028*	
N5	0.44272 (15)	0.73101 (11)	0.19350 (11)	0.0252 (4)	
H5A	0.4120	0.7140	0.1569	0.030*	
N6	0.50621 (15)	0.81663 (11)	0.25280 (10)	0.0227 (4)	
H6A	0.5238	0.8650	0.2618	0.027*	
N7	0.25878 (15)	0.93287 (11)	0.15208 (10)	0.0218 (3)	
N8	0.32617 (16)	0.54937 (12)	0.12943 (12)	0.0307 (4)	
N9	−0.08945 (16)	0.46810 (12)	0.22038 (12)	0.0310 (4)	
N10	−0.08201 (16)	0.77711 (12)	0.22959 (11)	0.0285 (4)	
N11	0.50651 (16)	0.16298 (12)	0.10090 (11)	0.0270 (4)	
N12	0.5052 (2)	0.29939 (14)	0.33842 (13)	0.0414 (5)	
N13	0.55052 (16)	−0.03116 (12)	0.39430 (11)	0.0297 (4)	
N14	0.87694 (16)	0.92056 (12)	0.45101 (11)	0.0280 (4)	
N15	0.8432 (2)	0.60241 (14)	0.53516 (15)	0.0453 (5)	
N16	0.79938 (16)	0.76219 (11)	0.76568 (11)	0.0279 (4)	
N18	0.9138 (2)	0.42750 (16)	0.01524 (15)	0.0541 (6)	
N17	0.76021 (19)	0.81449 (14)	0.08431 (13)	0.0401 (5)	
C1	0.18632 (18)	0.75699 (13)	0.39846 (13)	0.0256 (4)	
C2	0.1757 (2)	0.66653 (15)	0.43415 (15)	0.0335 (5)	
H2B	0.1705	0.6272	0.3995	0.040*	
C3	0.1730 (2)	0.63736 (16)	0.52277 (16)	0.0392 (6)	
H3B	0.1661	0.5760	0.5498	0.047*	
C4	0.1801 (2)	0.69480 (16)	0.57392 (15)	0.0401 (6)	
H4B	0.1780	0.6716	0.6347	0.048*	
C5	0.1901 (2)	0.78520 (15)	0.53837 (14)	0.0330 (5)	
H5B	0.1945	0.8247	0.5731	0.040*	
C6	0.19351 (18)	0.81461 (13)	0.44906 (13)	0.0247 (4)	
C7	0.20641 (17)	0.89165 (13)	0.31229 (12)	0.0212 (4)	
C8	0.22002 (19)	0.96737 (13)	0.23388 (12)	0.0252 (4)	
H8A	0.1362	1.0136	0.2346	0.030*	
H8B	0.2859	0.9983	0.2365	0.030*	
C9	0.2139 (2)	1.00563 (13)	0.08031 (13)	0.0259 (4)	
H9A	0.2794	1.0429	0.0527	0.031*	
H9B	0.1316	1.0469	0.1038	0.031*	
C10	0.19338 (17)	0.96346 (13)	0.01371 (12)	0.0221 (4)	
C11	0.16236 (17)	0.94336 (13)	−0.10929 (12)	0.0226 (4)	
C12	0.15096 (18)	0.95044 (15)	−0.19487 (13)	0.0285 (5)	
H12A	0.1647	1.0024	−0.2398	0.034*	
C13	0.1184 (2)	0.87699 (15)	−0.20989 (14)	0.0330 (5)	
H13A	0.1110	0.8780	−0.2673	0.040*	
C14	0.0957 (2)	0.80077 (16)	−0.14332 (15)	0.0356 (5)	
H14A	0.0725	0.7522	−0.1568	0.043*	
C15	0.1064 (2)	0.79473 (15)	−0.05901 (15)	0.0329 (5)	
H15A	0.0908	0.7433	−0.0138	0.040*	
C16	0.14117 (18)	0.86745 (14)	−0.04346 (13)	0.0251 (4)	
C17	0.40172 (18)	0.89956 (14)	0.12558 (13)	0.0248 (4)	
H17A	0.4444	0.9490	0.1223	0.030*	
H17B	0.4235	0.8847	0.0666	0.030*	
C18	0.45217 (17)	0.81625 (13)	0.18870 (12)	0.0225 (4)	
C19	0.53047 (18)	0.72800 (13)	0.30347 (13)	0.0239 (4)	
C20	0.58034 (19)	0.69227 (14)	0.37957 (13)	0.0295 (5)	
H20A	0.6085	0.7292	0.4058	0.035*	
C21	0.5861 (2)	0.60002 (15)	0.41413 (14)	0.0343 (5)	
H21A	0.6190	0.5727	0.4660	0.041*	
C22	0.5453 (2)	0.54537 (15)	0.37567 (15)	0.0360 (5)	
H22A	0.5520	0.4820	0.4019	0.043*	
C23	0.4956 (2)	0.58018 (14)	0.30116 (15)	0.0319 (5)	
H23A	0.4672	0.5429	0.2753	0.038*	
C24	0.48936 (18)	0.67315 (13)	0.26583 (13)	0.0251 (4)	
C25	0.13096 (18)	0.66971 (13)	0.18346 (12)	0.0233 (4)	
C26	0.18751 (18)	0.57814 (13)	0.16620 (13)	0.0238 (4)	
C27	0.11648 (19)	0.51360 (13)	0.17898 (13)	0.0254 (4)	
H27A	0.1585	0.4544	0.1659	0.030*	
C28	−0.01533 (18)	0.53544 (13)	0.21077 (13)	0.0257 (4)	
C29	−0.07911 (19)	0.62129 (14)	0.22969 (13)	0.0268 (4)	
H29A	−0.1701	0.6354	0.2526	0.032*	
C30	−0.00806 (19)	0.68566 (13)	0.21469 (12)	0.0247 (4)	
C31	0.53576 (17)	0.05662 (14)	0.24384 (13)	0.0233 (4)	
C32	0.51996 (18)	0.14913 (13)	0.19069 (13)	0.0235 (4)	
C33	0.51562 (18)	0.22584 (14)	0.21939 (13)	0.0273 (4)	
H33A	0.5091	0.2841	0.1806	0.033*	
C34	0.52086 (19)	0.21746 (14)	0.30585 (14)	0.0292 (5)	
C35	0.53357 (19)	0.13207 (14)	0.36288 (13)	0.0275 (4)	
H35A	0.5386	0.1266	0.4218	0.033*	
C36	0.53860 (18)	0.05687 (14)	0.33255 (13)	0.0247 (4)	
C37	0.82270 (18)	0.85297 (13)	0.61195 (13)	0.0249 (4)	
C38	0.85192 (18)	0.84327 (14)	0.52128 (13)	0.0247 (4)	
C39	0.85685 (19)	0.76357 (14)	0.49720 (14)	0.0294 (5)	
H39A	0.8738	0.7612	0.4369	0.035*	
C40	0.8371 (2)	0.68644 (14)	0.56079 (15)	0.0316 (5)	
C41	0.81635 (19)	0.68765 (14)	0.64873 (14)	0.0302 (5)	
H41A	0.8037	0.6342	0.6919	0.036*	
C42	0.81428 (18)	0.76644 (13)	0.67287 (13)	0.0248 (4)	
C43	0.7582 (2)	0.75207 (16)	0.06067 (14)	0.0324 (5)	
C44	0.7551 (3)	0.67206 (18)	0.03153 (18)	0.0490 (6)	
H44A	0.7095	0.6322	0.0801	0.059*	
H44B	0.8442	0.6380	0.0119	0.059*	
H44C	0.7097	0.6918	−0.0171	0.059*	
C45	0.8089 (3)	0.43038 (17)	0.05165 (17)	0.0432 (6)	
C46	0.6769 (3)	0.4302 (2)	0.0968 (2)	0.0670 (9)	
H46A	0.6485	0.3835	0.0806	0.080*	
H46B	0.6208	0.4910	0.0803	0.080*	
H46C	0.6717	0.4159	0.1602	0.080*	

### Atomic displacement parameters (Å^2^)

**Table:**

	*U*^11^	*U*^22^	*U*^33^	*U*^12^	*U*^13^	*U*^23^
O1	0.0332 (7)	0.0233 (7)	0.0316 (8)	−0.0105 (6)	−0.0115 (6)	−0.0050 (6)
O2	0.0371 (9)	0.0497 (11)	0.0906 (15)	−0.0239 (8)	0.0139 (9)	−0.0444 (10)
O3	0.0349 (10)	0.0360 (11)	0.157 (2)	−0.0079 (8)	0.0050 (11)	−0.0422 (13)
O4	0.0393 (8)	0.0234 (8)	0.0521 (10)	−0.0098 (7)	−0.0112 (7)	−0.0066 (7)
O5	0.0279 (9)	0.0421 (10)	0.0789 (13)	−0.0131 (7)	−0.0019 (8)	−0.0206 (9)
O6	0.0355 (8)	0.0348 (9)	0.0396 (9)	−0.0084 (7)	0.0026 (7)	−0.0145 (7)
O7	0.0425 (9)	0.0213 (8)	0.0445 (9)	−0.0076 (7)	−0.0006 (7)	−0.0020 (7)
O8	0.0380 (8)	0.0228 (7)	0.0291 (8)	−0.0099 (6)	−0.0071 (6)	−0.0082 (6)
O9	0.0554 (10)	0.0374 (9)	0.0324 (9)	−0.0202 (8)	−0.0164 (7)	−0.0070 (7)
O10	0.0469 (9)	0.0341 (9)	0.0279 (8)	−0.0176 (7)	−0.0045 (7)	0.0019 (7)
O11	0.138 (2)	0.0314 (10)	0.0469 (12)	−0.0357 (11)	−0.0051 (12)	−0.0114 (9)
O12	0.0904 (14)	0.0512 (12)	0.0391 (11)	−0.0256 (10)	−0.0138 (9)	−0.0215 (9)
O13	0.0340 (8)	0.0496 (10)	0.0343 (9)	−0.0025 (7)	−0.0144 (7)	0.0006 (7)
O14	0.0700 (11)	0.0304 (9)	0.0352 (9)	−0.0247 (8)	−0.0114 (8)	−0.0003 (7)
O15	0.0869 (13)	0.0222 (8)	0.0262 (8)	−0.0159 (8)	−0.0044 (8)	−0.0072 (7)
O16	0.0852 (13)	0.0315 (9)	0.0305 (9)	−0.0275 (9)	−0.0103 (8)	−0.0040 (7)
O17	0.0408 (8)	0.0415 (9)	0.0240 (8)	−0.0056 (7)	−0.0044 (6)	−0.0079 (7)
O18	0.0856 (14)	0.0467 (11)	0.0577 (13)	−0.0104 (10)	−0.0280 (10)	−0.0283 (9)
O19	0.1228 (19)	0.0317 (11)	0.0722 (15)	−0.0292 (11)	−0.0209 (13)	−0.0143 (10)
O20	0.0365 (8)	0.0282 (8)	0.0318 (8)	−0.0068 (6)	−0.0036 (6)	0.0002 (7)
O21	0.0603 (10)	0.0335 (9)	0.0351 (9)	−0.0157 (8)	−0.0197 (8)	−0.0058 (7)
N1	0.0267 (8)	0.0220 (9)	0.0225 (9)	−0.0063 (7)	−0.0028 (6)	−0.0076 (7)
N2	0.0294 (9)	0.0213 (9)	0.0229 (9)	−0.0079 (7)	−0.0051 (7)	−0.0067 (7)
N3	0.0319 (9)	0.0208 (9)	0.0227 (9)	−0.0085 (7)	−0.0085 (7)	−0.0026 (7)
N4	0.0274 (8)	0.0209 (8)	0.0227 (9)	−0.0065 (7)	−0.0066 (6)	−0.0023 (7)
N5	0.0271 (8)	0.0221 (9)	0.0301 (9)	−0.0044 (7)	−0.0073 (7)	−0.0118 (7)
N6	0.0265 (8)	0.0199 (8)	0.0244 (9)	−0.0080 (7)	−0.0055 (6)	−0.0059 (7)
N7	0.0276 (8)	0.0192 (8)	0.0200 (8)	−0.0049 (6)	−0.0066 (6)	−0.0052 (6)
N8	0.0304 (9)	0.0232 (10)	0.0411 (11)	−0.0080 (8)	−0.0077 (8)	−0.0085 (8)
N9	0.0320 (10)	0.0279 (10)	0.0337 (10)	−0.0125 (8)	−0.0048 (7)	−0.0033 (8)
N10	0.0320 (9)	0.0261 (10)	0.0287 (9)	−0.0060 (7)	−0.0071 (7)	−0.0079 (8)
N11	0.0295 (9)	0.0248 (9)	0.0261 (9)	−0.0058 (7)	−0.0048 (7)	−0.0054 (7)
N12	0.0591 (13)	0.0343 (11)	0.0383 (12)	−0.0207 (9)	−0.0038 (9)	−0.0158 (9)
N13	0.0311 (9)	0.0298 (10)	0.0247 (9)	−0.0045 (8)	−0.0039 (7)	−0.0048 (7)
N14	0.0297 (9)	0.0318 (10)	0.0248 (9)	−0.0079 (7)	−0.0085 (7)	−0.0059 (8)
N15	0.0623 (13)	0.0280 (11)	0.0541 (14)	−0.0095 (9)	−0.0227 (11)	−0.0131 (10)
N16	0.0312 (9)	0.0205 (9)	0.0295 (10)	−0.0035 (7)	−0.0074 (7)	−0.0026 (8)
N18	0.0538 (14)	0.0551 (15)	0.0544 (14)	−0.0180 (11)	−0.0075 (11)	−0.0109 (11)
N17	0.0497 (12)	0.0337 (11)	0.0376 (11)	−0.0103 (9)	−0.0118 (9)	−0.0047 (9)
C1	0.0240 (10)	0.0212 (10)	0.0296 (11)	−0.0045 (8)	−0.0023 (8)	−0.0061 (8)
C2	0.0342 (11)	0.0222 (11)	0.0408 (13)	−0.0068 (9)	−0.0025 (9)	−0.0058 (9)
C3	0.0418 (13)	0.0234 (11)	0.0420 (14)	−0.0072 (9)	−0.0030 (10)	0.0044 (10)
C4	0.0479 (14)	0.0352 (13)	0.0291 (12)	−0.0087 (10)	−0.0078 (10)	0.0061 (10)
C5	0.0419 (12)	0.0288 (12)	0.0256 (11)	−0.0081 (9)	−0.0070 (9)	−0.0011 (9)
C6	0.0259 (10)	0.0205 (10)	0.0254 (10)	−0.0066 (8)	−0.0037 (8)	−0.0012 (8)
C7	0.0213 (9)	0.0188 (10)	0.0230 (10)	−0.0045 (7)	−0.0026 (7)	−0.0059 (8)
C8	0.0332 (11)	0.0206 (10)	0.0222 (10)	−0.0053 (8)	−0.0050 (8)	−0.0069 (8)
C9	0.0345 (11)	0.0199 (10)	0.0241 (10)	−0.0055 (8)	−0.0097 (8)	−0.0028 (8)
C10	0.0226 (9)	0.0208 (10)	0.0224 (10)	−0.0036 (7)	−0.0064 (7)	−0.0034 (8)
C11	0.0226 (9)	0.0207 (10)	0.0238 (10)	−0.0029 (8)	−0.0066 (7)	−0.0043 (8)
C12	0.0278 (10)	0.0306 (11)	0.0246 (11)	0.0006 (8)	−0.0080 (8)	−0.0069 (9)
C13	0.0330 (11)	0.0372 (13)	0.0324 (12)	0.0007 (9)	−0.0142 (9)	−0.0155 (10)
C14	0.0406 (12)	0.0317 (12)	0.0427 (13)	−0.0061 (10)	−0.0178 (10)	−0.0147 (10)
C15	0.0379 (12)	0.0276 (11)	0.0374 (12)	−0.0086 (9)	−0.0131 (9)	−0.0069 (9)
C16	0.0260 (10)	0.0253 (10)	0.0260 (11)	−0.0043 (8)	−0.0093 (8)	−0.0063 (8)
C17	0.0283 (10)	0.0252 (10)	0.0220 (10)	−0.0079 (8)	−0.0042 (8)	−0.0059 (8)
C18	0.0215 (9)	0.0224 (10)	0.0239 (10)	−0.0050 (8)	−0.0021 (7)	−0.0080 (8)
C19	0.0234 (9)	0.0186 (10)	0.0267 (10)	−0.0042 (8)	−0.0016 (8)	−0.0041 (8)
C20	0.0308 (11)	0.0282 (11)	0.0286 (11)	−0.0069 (9)	−0.0059 (8)	−0.0048 (9)
C21	0.0350 (11)	0.0321 (12)	0.0296 (12)	−0.0042 (9)	−0.0060 (9)	−0.0004 (9)
C22	0.0381 (12)	0.0221 (11)	0.0397 (13)	−0.0035 (9)	−0.0027 (10)	−0.0023 (9)
C23	0.0327 (11)	0.0206 (11)	0.0418 (13)	−0.0053 (8)	−0.0036 (9)	−0.0104 (9)
C24	0.0219 (9)	0.0212 (10)	0.0312 (11)	−0.0026 (8)	−0.0026 (8)	−0.0093 (8)
C25	0.0310 (10)	0.0225 (10)	0.0186 (10)	−0.0091 (8)	−0.0098 (8)	0.0003 (8)
C26	0.0269 (10)	0.0209 (10)	0.0244 (10)	−0.0066 (8)	−0.0078 (8)	−0.0023 (8)
C27	0.0334 (11)	0.0177 (10)	0.0260 (11)	−0.0065 (8)	−0.0100 (8)	−0.0013 (8)
C28	0.0305 (10)	0.0215 (10)	0.0270 (11)	−0.0115 (8)	−0.0070 (8)	−0.0010 (8)
C29	0.0291 (10)	0.0268 (11)	0.0243 (10)	−0.0085 (8)	−0.0056 (8)	−0.0026 (8)
C30	0.0327 (10)	0.0191 (10)	0.0216 (10)	−0.0055 (8)	−0.0064 (8)	−0.0027 (8)
C31	0.0219 (9)	0.0244 (11)	0.0252 (10)	−0.0080 (8)	−0.0044 (7)	−0.0052 (8)
C32	0.0250 (10)	0.0228 (10)	0.0243 (10)	−0.0071 (8)	−0.0053 (7)	−0.0056 (8)
C33	0.0279 (10)	0.0240 (11)	0.0304 (11)	−0.0089 (8)	−0.0044 (8)	−0.0048 (9)
C34	0.0338 (11)	0.0274 (11)	0.0315 (11)	−0.0123 (9)	−0.0045 (8)	−0.0113 (9)
C35	0.0292 (10)	0.0331 (12)	0.0249 (10)	−0.0121 (9)	−0.0069 (8)	−0.0071 (9)
C36	0.0240 (10)	0.0246 (10)	0.0259 (11)	−0.0076 (8)	−0.0054 (8)	−0.0036 (8)
C37	0.0274 (10)	0.0205 (10)	0.0277 (11)	−0.0065 (8)	−0.0051 (8)	−0.0062 (8)
C38	0.0241 (10)	0.0242 (10)	0.0279 (11)	−0.0045 (8)	−0.0090 (8)	−0.0063 (8)
C39	0.0309 (11)	0.0312 (12)	0.0301 (11)	−0.0037 (9)	−0.0121 (8)	−0.0109 (9)
C40	0.0368 (11)	0.0223 (11)	0.0412 (13)	−0.0057 (9)	−0.0151 (9)	−0.0101 (9)
C41	0.0318 (11)	0.0232 (11)	0.0366 (12)	−0.0056 (8)	−0.0120 (9)	−0.0035 (9)
C42	0.0256 (10)	0.0208 (10)	0.0284 (11)	−0.0031 (8)	−0.0080 (8)	−0.0060 (8)
C43	0.0367 (12)	0.0293 (12)	0.0292 (12)	−0.0066 (9)	−0.0083 (9)	−0.0022 (10)
C44	0.0597 (16)	0.0386 (14)	0.0520 (16)	−0.0052 (12)	−0.0166 (12)	−0.0166 (12)
C45	0.0488 (15)	0.0367 (14)	0.0475 (15)	−0.0114 (11)	−0.0126 (12)	−0.0099 (11)
C46	0.0474 (16)	0.071 (2)	0.086 (2)	−0.0178 (15)	−0.0045 (15)	−0.0270 (18)

### Geometric parameters (Å, º)

**Table:**

O1—C25	1.268 (2)	C5—H5B	0.9500
O2—N8	1.206 (2)	C7—C8	1.491 (3)
O3—N8	1.215 (2)	C8—H8A	0.9900
O4—N9	1.224 (2)	C8—H8B	0.9900
O5—N9	1.228 (2)	C9—C10	1.490 (3)
O6—N10	1.227 (2)	C9—H9A	0.9900
O7—N10	1.230 (2)	C9—H9B	0.9900
O8—C31	1.250 (2)	C11—C16	1.391 (3)
O9—N11	1.228 (2)	C11—C12	1.397 (3)
O10—N11	1.230 (2)	C12—C13	1.379 (3)
O11—N12	1.232 (3)	C12—H12A	0.9500
O12—N12	1.218 (3)	C13—C14	1.404 (3)
O13—N13	1.230 (2)	C13—H13A	0.9500
O14—N13	1.227 (2)	C14—C15	1.377 (3)
O15—C37	1.236 (2)	C14—H14A	0.9500
O16—N14	1.222 (2)	C15—C16	1.386 (3)
O17—N14	1.235 (2)	C15—H15A	0.9500
O18—N15	1.227 (3)	C17—C18	1.489 (3)
O19—N15	1.231 (3)	C17—H17A	0.9900
O20—N16	1.243 (2)	C17—H17B	0.9900
O21—N16	1.224 (2)	C19—C24	1.390 (3)
N1—C7	1.332 (2)	C19—C20	1.398 (3)
N1—C1	1.390 (3)	C20—C21	1.379 (3)
N1—H1A	0.8800	C20—H20A	0.9500
N2—C7	1.325 (2)	C21—C22	1.395 (3)
N2—C6	1.389 (3)	C21—H21A	0.9500
N2—H2A	0.8800	C22—C23	1.373 (3)
N3—C10	1.328 (3)	C22—H22A	0.9500
N3—C16	1.388 (3)	C23—C24	1.390 (3)
N3—H3A	0.8800	C23—H23A	0.9500
N4—C10	1.325 (2)	C25—C26	1.441 (3)
N4—C11	1.395 (3)	C25—C30	1.443 (3)
N4—H4A	0.8800	C26—C27	1.379 (3)
N5—C18	1.332 (3)	C27—C28	1.373 (3)
N5—C24	1.394 (3)	C27—H27A	0.9500
N5—H5A	0.8800	C28—C29	1.382 (3)
N6—C18	1.329 (2)	C29—C30	1.370 (3)
N6—C19	1.393 (2)	C29—H29A	0.9500
N6—H6A	0.8800	C31—C36	1.445 (3)
N7—C8	1.467 (2)	C31—C32	1.452 (3)
N7—C9	1.471 (2)	C32—C33	1.367 (3)
N7—C17	1.485 (2)	C33—C34	1.386 (3)
N8—C26	1.461 (3)	C33—H33A	0.9500
N9—C28	1.451 (3)	C34—C35	1.393 (3)
N10—C30	1.459 (3)	C35—C36	1.357 (3)
N11—C32	1.453 (3)	C35—H35A	0.9500
N12—C34	1.445 (3)	C37—C42	1.450 (3)
N13—C36	1.457 (3)	C37—C38	1.453 (3)
N14—C38	1.451 (3)	C38—C39	1.369 (3)
N15—C40	1.442 (3)	C39—C40	1.383 (3)
N16—C42	1.452 (3)	C39—H39A	0.9500
N18—C45	1.139 (3)	C40—C41	1.383 (3)
N17—C43	1.135 (3)	C41—C42	1.366 (3)
C1—C6	1.388 (3)	C41—H41A	0.9500
C1—C2	1.392 (3)	C43—C44	1.446 (3)
C2—C3	1.380 (3)	C44—H44A	0.9800
C2—H2B	0.9500	C44—H44B	0.9800
C3—C4	1.393 (4)	C44—H44C	0.9800
C3—H3B	0.9500	C45—C46	1.437 (4)
C4—C5	1.389 (3)	C46—H46A	0.9800
C4—H4B	0.9500	C46—H46B	0.9800
C5—C6	1.390 (3)	C46—H46C	0.9800
			
C7—N1—C1	108.92 (16)	C15—C16—N3	131.52 (19)
C7—N1—H1A	125.5	C15—C16—C11	121.77 (18)
C1—N1—H1A	125.5	N3—C16—C11	106.72 (17)
C7—N2—C6	108.99 (16)	N7—C17—C18	111.47 (15)
C7—N2—H2A	125.5	N7—C17—H17A	109.3
C6—N2—H2A	125.5	C18—C17—H17A	109.3
C10—N3—C16	108.85 (16)	N7—C17—H17B	109.3
C10—N3—H3A	125.6	C18—C17—H17B	109.3
C16—N3—H3A	125.6	H17A—C17—H17B	108.0
C10—N4—C11	109.27 (16)	N6—C18—N5	109.41 (16)
C10—N4—H4A	125.4	N6—C18—C17	124.92 (18)
C11—N4—H4A	125.4	N5—C18—C17	125.54 (17)
C18—N5—C24	109.02 (16)	C24—C19—N6	106.44 (17)
C18—N5—H5A	125.5	C24—C19—C20	121.51 (18)
C24—N5—H5A	125.5	N6—C19—C20	132.01 (18)
C18—N6—C19	108.98 (16)	C21—C20—C19	115.8 (2)
C18—N6—H6A	125.5	C21—C20—H20A	122.1
C19—N6—H6A	125.5	C19—C20—H20A	122.1
C8—N7—C9	110.46 (15)	C20—C21—C22	122.3 (2)
C8—N7—C17	112.55 (15)	C20—C21—H21A	118.8
C9—N7—C17	108.15 (15)	C22—C21—H21A	118.8
O2—N8—O3	120.98 (19)	C23—C22—C21	122.1 (2)
O2—N8—C26	120.79 (17)	C23—C22—H22A	118.9
O3—N8—C26	118.13 (17)	C21—C22—H22A	118.9
O4—N9—O5	123.34 (18)	C22—C23—C24	116.0 (2)
O4—N9—C28	118.64 (17)	C22—C23—H23A	122.0
O5—N9—C28	118.02 (17)	C24—C23—H23A	122.0
O6—N10—O7	123.04 (17)	C23—C24—C19	122.25 (19)
O6—N10—C30	118.43 (17)	C23—C24—N5	131.61 (19)
O7—N10—C30	118.48 (16)	C19—C24—N5	106.12 (17)
O9—N11—O10	122.66 (17)	O1—C25—C26	124.49 (18)
O9—N11—C32	118.81 (17)	O1—C25—C30	123.50 (18)
O10—N11—C32	118.51 (17)	C26—C25—C30	112.01 (17)
O12—N12—O11	123.1 (2)	C27—C26—C25	123.59 (18)
O12—N12—C34	119.0 (2)	C27—C26—N8	115.42 (17)
O11—N12—C34	117.9 (2)	C25—C26—N8	120.93 (17)
O14—N13—O13	123.36 (18)	C28—C27—C26	119.60 (18)
O14—N13—C36	118.97 (16)	C28—C27—H27A	120.2
O13—N13—C36	117.64 (17)	C26—C27—H27A	120.2
O16—N14—O17	121.47 (18)	C27—C28—C29	121.43 (18)
O16—N14—C38	120.31 (17)	C27—C28—N9	118.90 (18)
O17—N14—C38	118.22 (18)	C29—C28—N9	119.60 (18)
O18—N15—O19	123.1 (2)	C30—C29—C28	118.48 (18)
O18—N15—C40	119.1 (2)	C30—C29—H29A	120.8
O19—N15—C40	117.8 (2)	C28—C29—H29A	120.8
O21—N16—O20	122.63 (17)	C29—C30—C25	124.84 (18)
O21—N16—C42	119.46 (17)	C29—C30—N10	115.98 (17)
O20—N16—C42	117.90 (17)	C25—C30—N10	119.15 (17)
C6—C1—N1	106.17 (17)	O8—C31—C36	124.85 (18)
C6—C1—C2	121.96 (19)	O8—C31—C32	123.78 (18)
N1—C1—C2	131.86 (19)	C36—C31—C32	111.21 (17)
C3—C2—C1	116.1 (2)	C33—C32—C31	124.46 (18)
C3—C2—H2B	121.9	C33—C32—N11	116.66 (18)
C1—C2—H2B	121.9	C31—C32—N11	118.89 (17)
C2—C3—C4	122.2 (2)	C32—C33—C34	119.12 (19)
C2—C3—H3B	118.9	C32—C33—H33A	120.4
C4—C3—H3B	118.9	C34—C33—H33A	120.4
C5—C4—C3	121.7 (2)	C33—C34—C35	121.02 (19)
C5—C4—H4B	119.2	C33—C34—N12	119.27 (19)
C3—C4—H4B	119.2	C35—C34—N12	119.60 (19)
C4—C5—C6	116.2 (2)	C36—C35—C34	118.66 (19)
C4—C5—H5B	121.9	C36—C35—H35A	120.7
C6—C5—H5B	121.9	C34—C35—H35A	120.7
C1—C6—N2	106.46 (17)	C35—C36—C31	125.44 (19)
C1—C6—C5	121.80 (19)	C35—C36—N13	117.15 (18)
N2—C6—C5	131.72 (19)	C31—C36—N13	117.40 (18)
N2—C7—N1	109.46 (16)	O15—C37—C42	122.38 (19)
N2—C7—C8	124.27 (17)	O15—C37—C38	125.31 (19)
N1—C7—C8	126.27 (17)	C42—C37—C38	112.23 (17)
N7—C8—C7	111.74 (16)	C39—C38—N14	116.76 (18)
N7—C8—H8A	109.3	C39—C38—C37	123.26 (19)
C7—C8—H8A	109.3	N14—C38—C37	119.98 (18)
N7—C8—H8B	109.3	C38—C39—C40	119.9 (2)
C7—C8—H8B	109.3	C38—C39—H39A	120.1
H8A—C8—H8B	107.9	C40—C39—H39A	120.1
N7—C9—C10	109.82 (15)	C41—C40—C39	120.9 (2)
N7—C9—H9A	109.7	C41—C40—N15	119.4 (2)
C10—C9—H9A	109.7	C39—C40—N15	119.7 (2)
N7—C9—H9B	109.7	C42—C41—C40	119.4 (2)
C10—C9—H9B	109.7	C42—C41—H41A	120.3
H9A—C9—H9B	108.2	C40—C41—H41A	120.3
N4—C10—N3	109.49 (17)	C41—C42—C37	123.93 (19)
N4—C10—C9	126.45 (17)	C41—C42—N16	116.95 (18)
N3—C10—C9	123.92 (17)	C37—C42—N16	119.10 (17)
C16—C11—N4	105.67 (16)	N17—C43—C44	179.4 (3)
C16—C11—C12	122.08 (19)	C43—C44—H44A	109.5
N4—C11—C12	132.25 (19)	C43—C44—H44B	109.5
C13—C12—C11	115.63 (19)	H44A—C44—H44B	109.5
C13—C12—H12A	122.2	C43—C44—H44C	109.5
C11—C12—H12A	122.2	H44A—C44—H44C	109.5
C12—C13—C14	122.3 (2)	H44B—C44—H44C	109.5
C12—C13—H13A	118.8	N18—C45—C46	177.5 (3)
C14—C13—H13A	118.8	C45—C46—H46A	109.5
C15—C14—C13	121.6 (2)	C45—C46—H46B	109.5
C15—C14—H14A	119.2	H46A—C46—H46B	109.5
C13—C14—H14A	119.2	C45—C46—H46C	109.5
C14—C15—C16	116.6 (2)	H46A—C46—H46C	109.5
C14—C15—H15A	121.7	H46B—C46—H46C	109.5
C16—C15—H15A	121.7		
			
C7—N1—C1—C6	0.8 (2)	C25—C26—C27—C28	−0.8 (3)
C7—N1—C1—C2	−178.4 (2)	N8—C26—C27—C28	−178.02 (17)
C6—C1—C2—C3	−0.2 (3)	C26—C27—C28—C29	0.6 (3)
N1—C1—C2—C3	178.92 (19)	C26—C27—C28—N9	177.45 (17)
C1—C2—C3—C4	0.2 (3)	O4—N9—C28—C27	13.9 (3)
C2—C3—C4—C5	0.1 (4)	O5—N9—C28—C27	−166.01 (19)
C3—C4—C5—C6	−0.4 (3)	O4—N9—C28—C29	−169.14 (19)
N1—C1—C6—N2	−0.6 (2)	O5—N9—C28—C29	10.9 (3)
C2—C1—C6—N2	178.72 (17)	C27—C28—C29—C30	1.0 (3)
N1—C1—C6—C5	−179.45 (18)	N9—C28—C29—C30	−175.83 (17)
C2—C1—C6—C5	−0.2 (3)	C28—C29—C30—C25	−2.6 (3)
C7—N2—C6—C1	0.1 (2)	C28—C29—C30—N10	175.52 (17)
C7—N2—C6—C5	178.8 (2)	O1—C25—C30—C29	−177.11 (18)
C4—C5—C6—C1	0.4 (3)	C26—C25—C30—C29	2.3 (3)
C4—C5—C6—N2	−178.1 (2)	O1—C25—C30—N10	4.8 (3)
C6—N2—C7—N1	0.4 (2)	C26—C25—C30—N10	−175.78 (16)
C6—N2—C7—C8	−179.19 (17)	O6—N10—C30—C29	29.4 (3)
C1—N1—C7—N2	−0.8 (2)	O7—N10—C30—C29	−148.04 (18)
C1—N1—C7—C8	178.82 (17)	O6—N10—C30—C25	−152.36 (18)
C9—N7—C8—C7	153.47 (16)	O7—N10—C30—C25	30.2 (3)
C17—N7—C8—C7	−85.54 (19)	O8—C31—C32—C33	−172.20 (18)
N2—C7—C8—N7	161.60 (16)	C36—C31—C32—C33	3.4 (3)
N1—C7—C8—N7	−17.9 (3)	O8—C31—C32—N11	8.4 (3)
C8—N7—C9—C10	−152.31 (16)	C36—C31—C32—N11	−176.04 (16)
C17—N7—C9—C10	84.12 (19)	O9—N11—C32—C33	−145.85 (18)
C11—N4—C10—N3	0.2 (2)	O10—N11—C32—C33	32.6 (2)
C11—N4—C10—C9	−175.74 (17)	O9—N11—C32—C31	33.6 (2)
C16—N3—C10—N4	−0.3 (2)	O10—N11—C32—C31	−147.96 (17)
C16—N3—C10—C9	175.73 (17)	C31—C32—C33—C34	−2.9 (3)
N7—C9—C10—N4	−156.56 (17)	N11—C32—C33—C34	176.53 (17)
N7—C9—C10—N3	28.1 (2)	C32—C33—C34—C35	1.6 (3)
C10—N4—C11—C16	0.0 (2)	C32—C33—C34—N12	−174.76 (18)
C10—N4—C11—C12	−179.49 (19)	O12—N12—C34—C33	175.1 (2)
C16—C11—C12—C13	−0.5 (3)	O11—N12—C34—C33	−3.3 (3)
N4—C11—C12—C13	178.98 (19)	O12—N12—C34—C35	−1.3 (3)
C11—C12—C13—C14	1.1 (3)	O11—N12—C34—C35	−179.7 (2)
C12—C13—C14—C15	−0.8 (3)	C33—C34—C35—C36	−1.1 (3)
C13—C14—C15—C16	−0.3 (3)	N12—C34—C35—C36	175.22 (18)
C14—C15—C16—N3	−179.0 (2)	C34—C35—C36—C31	2.0 (3)
C14—C15—C16—C11	0.9 (3)	C34—C35—C36—N13	−179.36 (17)
C10—N3—C16—C15	−179.8 (2)	O8—C31—C36—C35	172.61 (19)
C10—N3—C16—C11	0.3 (2)	C32—C31—C36—C35	−2.9 (3)
N4—C11—C16—C15	179.87 (17)	O8—C31—C36—N13	−6.1 (3)
C12—C11—C16—C15	−0.5 (3)	C32—C31—C36—N13	178.39 (16)
N4—C11—C16—N3	−0.2 (2)	O14—N13—C36—C35	142.79 (19)
C12—C11—C16—N3	179.38 (17)	O13—N13—C36—C35	−35.4 (3)
C8—N7—C17—C18	66.3 (2)	O14—N13—C36—C31	−38.4 (2)
C9—N7—C17—C18	−171.37 (16)	O13—N13—C36—C31	143.43 (18)
C19—N6—C18—N5	−1.6 (2)	O16—N14—C38—C39	172.89 (18)
C19—N6—C18—C17	174.45 (17)	O17—N14—C38—C39	−7.9 (3)
C24—N5—C18—N6	1.8 (2)	O16—N14—C38—C37	−6.8 (3)
C24—N5—C18—C17	−174.16 (17)	O17—N14—C38—C37	172.39 (17)
N7—C17—C18—N6	−98.3 (2)	O15—C37—C38—C39	−170.2 (2)
N7—C17—C18—N5	77.0 (2)	C42—C37—C38—C39	6.5 (3)
C18—N6—C19—C24	0.7 (2)	O15—C37—C38—N14	9.4 (3)
C18—N6—C19—C20	−177.0 (2)	C42—C37—C38—N14	−173.81 (16)
C24—C19—C20—C21	0.1 (3)	N14—C38—C39—C40	178.44 (17)
N6—C19—C20—C21	177.5 (2)	C37—C38—C39—C40	−1.9 (3)
C19—C20—C21—C22	0.2 (3)	C38—C39—C40—C41	−2.1 (3)
C20—C21—C22—C23	−0.6 (3)	C38—C39—C40—N15	−179.58 (18)
C21—C22—C23—C24	0.5 (3)	O18—N15—C40—C41	−175.1 (2)
C22—C23—C24—C19	−0.1 (3)	O19—N15—C40—C41	5.0 (3)
C22—C23—C24—N5	−178.2 (2)	O18—N15—C40—C39	2.5 (3)
N6—C19—C24—C23	−178.12 (17)	O19—N15—C40—C39	−177.5 (2)
C20—C19—C24—C23	−0.2 (3)	C39—C40—C41—C42	0.6 (3)
N6—C19—C24—N5	0.4 (2)	N15—C40—C41—C42	178.11 (19)
C20—C19—C24—N5	178.35 (17)	C40—C41—C42—C37	5.0 (3)
C18—N5—C24—C23	177.0 (2)	C40—C41—C42—N16	−176.66 (17)
C18—N5—C24—C19	−1.3 (2)	O15—C37—C42—C41	168.7 (2)
O1—C25—C26—C27	178.83 (18)	C38—C37—C42—C41	−8.1 (3)
C30—C25—C26—C27	−0.6 (3)	O15—C37—C42—N16	−9.6 (3)
O1—C25—C26—N8	−4.1 (3)	C38—C37—C42—N16	173.53 (16)
C30—C25—C26—N8	176.56 (16)	O21—N16—C42—C41	153.01 (18)
O2—N8—C26—C27	−179.8 (2)	O20—N16—C42—C41	−25.9 (2)
O3—N8—C26—C27	3.8 (3)	O21—N16—C42—C37	−28.5 (3)
O2—N8—C26—C25	2.9 (3)	O20—N16—C42—C37	152.55 (17)
O3—N8—C26—C25	−173.5 (2)		

### Hydrogen-bond geometry (Å, º)

**Table:**

*D*—H···*A*	*D*—H	H···*A*	*D*···*A*	*D*—H···*A*
N1—H1*A*···O1	0.88	1.96	2.834 (2)	173
N2—H2*A*···O15^i^	0.88	1.88	2.619 (2)	140
N2—H2*A*···O16^i^	0.88	2.27	2.952 (2)	134
N3—H3*A*···O1	0.88	2.01	2.847 (2)	158
N3—H3*A*···O7	0.88	2.33	2.905 (2)	123
N4—H4*A*···N17^ii^	0.88	2.18	2.965 (3)	148
N5—H5*A*···O1	0.88	2.28	2.836 (2)	121
N5—H5*A*···O2	0.88	2.09	2.853 (2)	144
N6—H6*A*···O8^iii^	0.88	1.91	2.693 (2)	147
N6—H6*A*···O14^iii^	0.88	2.30	2.945 (2)	130

Symmetry codes: (i) −*x*+1, −*y*+2, −*z*+1; (ii) −*x*+1, −*y*+2, −*z*; (iii) *x*, *y*+1, *z*.
